# Control the source: Source memory for semantic, spatial and self-related items in patients with LIFG lesions

**DOI:** 10.1016/j.cortex.2019.04.014

**Published:** 2019-10

**Authors:** Sara Stampacchia, Suzanne Pegg, Glyn Hallam, Jonathan Smallwood, Matthew A. Lambon Ralph, Hannah Thompson, Elizabeth Jefferies

**Affiliations:** a Department of Psychology, University of York, UK; b Department of Psychology, School of Human and Health Sciences, University of Huddersfield, Huddersfield, UK; c MRC Cognition and Brain Sciences Unit, Cambridge, UK; d School of Psychology, University of Surrey, Guildford, UK

**Keywords:** Source memory, LIFG, Spatial processing, Self-reference effect, Semantic

## Abstract

Patients with multimodal semantic deficits following stroke (‘semantic aphasia’) have largely intact knowledge, yet difficulty controlling conceptual retrieval to suit the circumstances. Although conceptual representations are thought to be largely distinct from episodic representations of recent events, controlled retrieval processes may overlap across semantic and episodic memory domains. We investigated this possibility by examining item familiarity and source memory for recent events in semantic aphasia following infarcts affecting left inferior frontal gyrus. We tested the hypothesis that the nature of impairment in episodic judgements reflects the need for control over retrieval: item familiarity might be relatively intact, given it is driven by strong cues (re-presentation of the item), while source recollection might be more impaired since this task involves resolving competition between several potential sources. This pattern was observed most strongly when the degree of competition between sources was higher, i.e., when non-meaningful sources had similar perceptual features, and existing knowledge was incongruent with the source. In contrast, when (i) spatial location acted as a strong cue for retrieval; (ii) existing knowledge was congruent with episodic memory and (iii) distinctiveness of sources was increased by means of self-referential processing, source memory reached normal levels. These findings confirm the association between deregulated control of semantic and episodic memory in patients with semantic aphasia and delineate circumstances that ameliorate or aggravate these deficits.

## 1 Introduction

The retrieval of episodic memory is thought to result from an interplay between stored representations and control processes (Badre and Wagner, 2007, Levy and Anderson, 2002). A similar interaction between conceptual representations and control processes is thought to be critical in semantic cognition (cf. Controlled Semantic Cognition framework, Jefferies, 2013, Lambon Ralph et al., 2017). Moreover, while *representations* of conceptual and episodic memory are thought to be distinct, as reflected by clear neuropsychological dissociations (Manns et al., 2003, McKinnon et al., 2006, Nestor et al., 2006, Vargha-Khadem et al., 1997, Verfaellie et al., 2000), control processes that support the capacity to focus retrieval on currently-relevant memory representations may be shared across episodic and semantic tasks (Badre and Wagner, 2007, Burianova and Grady, 2007, Burianova et al., 2010, Rajah and McIntosh, 2005). This prediction emerges from neuroimaging studies of healthy participants that reveal activation in similar brain areas (including left inferior frontal gyrus, LIFG) during both semantic and episodic retrieval (Badre and Wagner, 2007, Burianova and Grady, 2007). However, few (if any) neuropsychological studies have examined semantic and episodic tasks in the same participants, and neuroimaging studies that have observed overlapping patterns of activation in LIFG are unable to determine if this region is necessary for performance on both of these tasks. Studies of the retrieval deficits of patients with LIFG lesions are especially useful in this context.

**Fig. 1:**
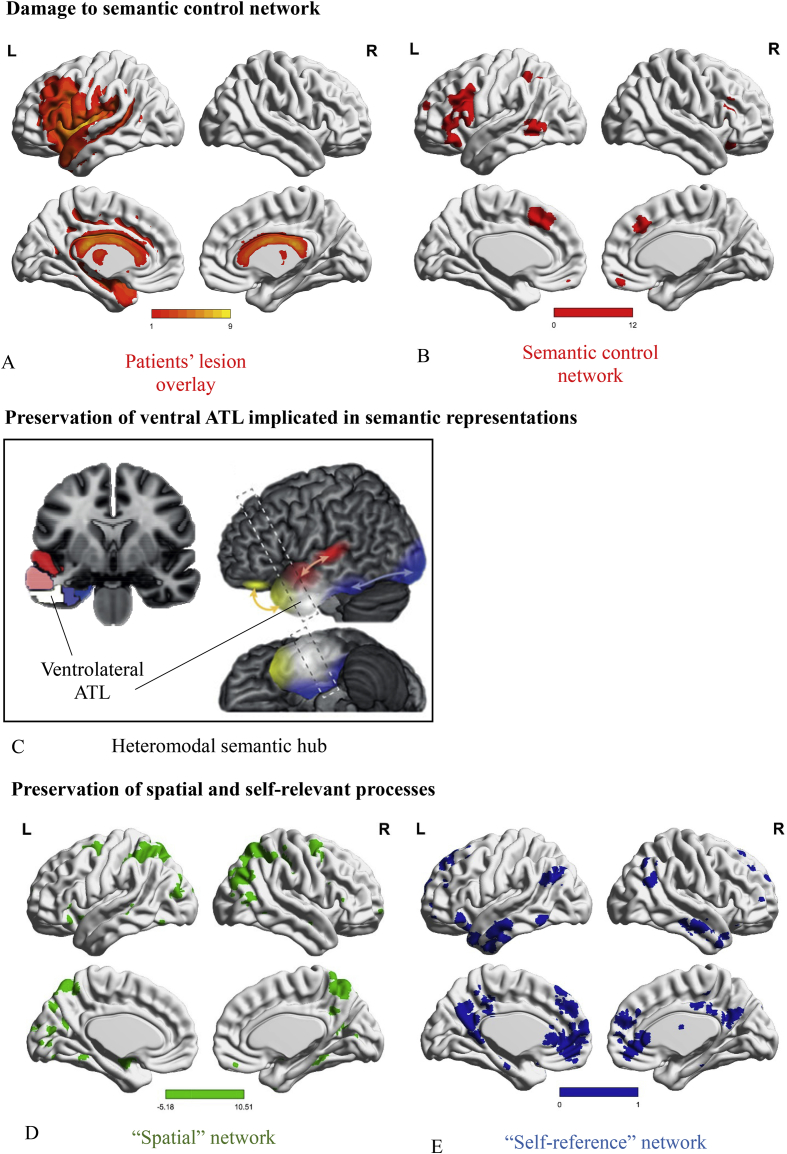
A) Lesion overlay of the sample of SA patients included in the study. Patients' brains compared to aged-matched controls. Grey matter, white matter and CSF were segmented and changes from the healthy control brains were highlighted as ‘lesion’ using automated methods (Seghier, Ramlackhansingh, Crinion, Leff, & Price, 2008). Colour bar indicates amount of overlap from 1 to 9 patients. B) Semantic control network from Noonan et al. (2013), adapted by Humphreys and Lambon Ralph (2015). C) Neuroanatomical sketch of the graded division within ATL in lateral and coronal cross-section views, adapted from Lambon Ralph et al. (2017) with permission. ATL subregions respond differentially to input sources: valence (yellow), audition (red) and vision (blue), while ventrolateral ATL (white) is equally engaged by all input types. It is proposed that ventrolateral ATL constitutes a heteromodal hub for semantic representation. D) Spatial network generated using Neurosynth: a meta-analysis of 1157 studies containing the term “spatial”. E) Self-reference network generated using Neurosynth: a meta-analysis of 127 studies containing the term “self-referential”. A, B, D and E were visualized with the BrainNet Viewer (Xia, Wang, & He, 2013, http://www.nitrc.org/projects/bnv/).

In a recent study, we investigated whether stroke aphasia patients with multimodal semantic impairment (i.e., semantic aphasia, SA) exhibited parallel deficits in semantic and episodic memory following infarcts in LIFG (Stampacchia et al., 2018). In line with preservation of ventrolateral portions of the anterior temporal lobes (ATL, see Fig, 1C) – a brain region which has been suggested to act as heteromodal hub of semantic knowledge (Binney et al., 2010, Lambon Ralph et al., 2017, Visser et al., 2012) – SA patients have largely intact conceptual knowledge but difficulty flexibly retrieving relevant information to suit the circumstances. These patients show inconsistent performance across tasks probing the same concepts but with differing control demands (Jefferies & Lambon Ralph, 2006). They are particularly impaired in understanding the subordinate meanings of words and non-canonical uses of objects (Corbett, Jefferies, & Lambon Ralph, 2011; Noonan, Jefferies, Corbett, & Lambon Ralph, 2010); they are sensitive to cues/miscues that direct or misdirect retrieval, and fail to inhibit strong yet irrelevant semantic distractors (Corbett et al., 2011, Jefferies et al., 2008, Noonan et al., 2010, Soni et al., 2009). These deficits are thought to reflect poor semantic control, i.e., the capacity to flexibly shape conceptual retrieval in an appropriate way. Accordingly, patients' lesions encompass areas known to support semantic control (according to a neuroimaging meta-analysis by Noonan, Jefferies, Visser, & Lambon Ralph, 2013, see Fig. 1A, B). This pattern of semantic impairment is qualitatively distinct from the degraded conceptual knowledge seen in semantic dementia (SD) following atrophy within ventral ATL, as SD patients show a high degree of consistency in which items are comprehended across tasks with differing demands (Jefferies and Lambon Ralph, 2006, Jefferies et al., 2008). Stampacchia et al. (2018) found that SA patients showed many of the hallmarks of deregulated retrieval in episodic as well as semantic decisions, using paired-associate tasks. Episodic judgements showed a benefit of cues that reduced the need to internally constrain retrieval. SA patients were vulnerable to strong but irrelevant semantic associates and previously-encoded associations-giving rise to false memories and proactive interference errors – and their episodic deficits were multimodal, affecting both word and picture tasks. These findings suggest that shared mechanisms underpin controlled retrieval from both semantic and episodic memory. However, Stampacchia et al. (2018) found some differences between verbal and non-verbal paired-associate learning tasks (e.g., reduced vulnerability to semantic and episodic interference for the picture-based episodic memory task) and it is unclear if this reflected modality-differences in memory control or task characteristics (it might be easier to reject picture distractors given the richness and distinctiveness of these stimuli). In the current study, we investigated: a) whether the episodic deficits found in SA would extend to other paradigms tapping episodic memory control; b) the multimodal nature of these deficits, using picture-based tasks; c) circumstances that could ameliorate or aggravate episodic deficits in SA.

We assessed whether the degree of episodic impairment in patients with SA varies across different types of memory judgement tapping item and source memory. In item memory, participants decide whether an item was previously encountered by means of an old/new response. In contrast, source memory tasks require participants to retrieve the circumstances in which an item was encoded – for example, the time, spatial context or task in which it was previously encountered. Ageing and neuropsychological studies show dissociations between item and source memory. Damage to medial-temporal structures affects both types of memory judgements (Dede et al., 2013, Gold et al., 2006), while frontal lobe damage is associated with source memory impairment and minimal or no item memory deficits (Janowsky et al., 1989, Schacter, 1987). Likewise, source memory declines in old age, while item memory is generally unaffected (Chalfonte and Johnson, 1996, Naveh-Benjamin, 2000).

Functional neuroimaging studies show that source memory engages medial-temporal structures (Ross & Slotnick, 2008) – but also a network of areas associated with controlled memory retrieval, including LIFG (Dobbins et al., 2002, Dobbins and Wagner, 2005, Han et al., 2012, Hayes et al., 2011). LIFG, which is thought to resolve competition between competing memories (Badre & Wagner, 2007), is more necessary in source memory compared to item memory for several reasons: (i) In item recognition, presentation of the item acts as a strong external cue reducing competition between memories; (ii) During source memory tasks, there are typically two or more alternative source options for each item, giving rise to competition. Differences in the nature of the sources – i.e., their distinctiveness and/or compatibility with previous knowledge or experience – should influence the degree of control required and the likelihood of potential source memory failures.

In this study, we compared item and source memory in a case-series of SA patients with deregulated semantic retrieval following infarcts that affected left lateral prefrontal cortex including LIFG. We predicted that source memory would show significant impairment in this group, but item memory would be largely unaffected. We also expected source memory deficits to be ameliorated by the presentation of strong cues that distinguished between otherwise confusable sources, but worsened in circumstances that increase competition between sources. The degree of competition was manipulated in three ways. (i) First, we expected spatial location to act as a strong cue for retrieval (Robin and Moscovitch, 2014, Smith et al., 2014), since the network supporting spatial representations is largely intact in patients with SA (see Fig. 1D). A match in spatial location between encoding and retrieval should reduce the demands on controlled retrieval, since it provides a potent cue to separate sources. (ii) We also expected better performance when existing knowledge was congruent with episodic memory. Previous research has demonstrated semantic cueing improves comprehension of ambiguous words in SA (Corbett et al., 2011, Noonan et al., 2010). Here we expected patients to show reduced source memory impairment when sources were congruent with pre-existing knowledge. Conversely, source memory deficits should be magnified when a source competes with existing knowledge (e.g., when a carrot was located in a clothes shop, not a greengrocer). (iii) Finally, we expected deficits to be reduced when the distinctiveness of sources was increased by means of self-referential processing. Self-referenced items are typically better recalled because they are more meaningful and distinctive (Dulas et al., 2011, Durbin et al., 2017, Hamami et al., 2011, Rosa and Gutchess, 2011, Serbun et al., 2011) – and this might reduce competition between sources. Self-reference effects have been linked to regions including medial prefrontal cortex (De Caso et al., 2017, Kelley et al., 2002, Macrae et al., 2004, Wong et al., 2017) that are largely intact in semantic aphasia. In summary, this study examined whether patients with semantic aphasia have an episodic memory deficit that is linked to poor control over memory retrieval beyond the semantic and language domain, using non-verbal source memory tasks, and investigated factors that ameliorate or aggravate these deficits.

## 2 Participants

### 2.1 Patients

Nine participants [5 female; age range 40–78, M = 63 years (SD = 11.5); mean education leaving age = 16.4 years (SD = 1.2); mean years since CVA = 8.8 (SD = 5.9)] with chronic stroke aphasia from left-hemisphere CVA were recruited from communication groups in Yorkshire, UK. The patients were selected to have multimodal semantic deficits. We recruited the sample reported by Stampacchia et al. (2018) although that study included one additional patient (referred to as P8), who was not available for testing in the current study. Sample size was determined by the maximum number of patients available for testing. These criteria for including participants were established prior to data collection. On the basis of their aphasic symptomatology, the patients could be classified as follows: two Global; two Mixed Transcortical; four Transcortical Sensory/Anomic; one Broca. One patient (P4) withdrew from the study part-way through and took part in Experiments 1 and 2 only. Individual data are provided in Supplementary Table 1.

#### 2.1.1 Inclusion criteria

In line with the original use of the term “semantic aphasia” by Henry Head (1926) and the inclusion criteria proposed by Jefferies and Lambon Ralph (2006), the patients in this study were selected to show deficits affecting the appropriate use of concepts presented as words and objects when control demands were high. In addition to verbal semantic problems, they were impaired on at least one non-verbal task (see section 3.2). There were no other inclusion/exclusion criteria. In common with previous SA samples, the patients showed strong effects of semantic control manipulations across tasks (details below). Individual patient data and task descriptions are provided in section 3.2.

#### 2.1.2 Lesion analysis

MRI scans were traced onto standardized templates (Damasio & Damasio, 1989) and lesion identification was manually performed (see Table 1 and Fig. 1A for lesion overlay). All nine patients had lesions affecting left posterior LIFG; in seven cases this damage extended to mid-to-anterior LIFG. Parietal regions (supramarginal gyrus and/or angular gyrus) were also affected in 7 cases out of 9, and pMTG was affected in all but two cases. While there was some damage to ATL in 3 patients (P1, P2, P4), the ventral portion of ATL, which has been implicated in conceptual representation across modalities (Binney et al., 2012, Visser et al., 2012), was intact in all cases. This region is supplied by both the anterior temporal cortical artery of the middle cerebral artery and the anterior temporal branch of the distal posterior cerebral artery, reducing its vulnerability to stroke (Borden, 2006, Conn, 2008, Phan et al., 2005). The hippocampus and parahippocampal gyrus were intact in all patients and medial PFC was also spared, although cingulate cortex was affected in two patients (P6 and P7).

**Table 1:** Patients' lesion analysis.

Patient ID	Lesion size*	Fronto-lateral							Medial				Parieto - temporal									
		SMA/PMC	FP	DLPFC		ant-IFG	mid-IFG	post-IFG	vm-PFC	dm-PFC	ACC	PCC	SMG	AnG	pMTG	STG	MTG	ITG	FuG	TP	PHG	Hpc
		Brodmann Areas																				
		6	10	9	46	47	45	44	10	9	24/32/33	23/31	40	39	37	22	21	20	36	38	28	28
P1	12	1		1	1		1	1					2	1	1	2				1		
P2	15	2		2		2	2	2					1			2						
P3	15	2		2		2	1	2					2	1	2	2	2	1				
P4	8	2						1					1		2							
P5	15	2						2				2	2	1	1							
P6	7	1					1	2			1	1	1	1	1							
P7	14	2				2	1	2			1	1	1	1	1	2	1					
P9	4	1						1							1	1	1					
P10	9	0					1	2								2						

*Note*. MRI scans were manually traced onto Damasio templates. Lesion size* was calculated as % template damaged. For areas not comprehensively characterized by Damasio templates, analyses were combined with manual analysis of the structural scan with the help of a trained radiographer. Quantification of lesion: 2 = complete destruction/serious damage to cortical grey matter; 1 = partial destruction/mild damage to cortical grey matter; empty = intact. Anatomical abbreviations: SMA/PMC: Supplementary Motor Area/Premotor Cortex; FP: Frontal Pole; DLPFC: Dorsolateral Prefrontal Cortex; ant-IFG: Inferior Frontal Gyrus, pars orbitalis; mid-IFG: Inferior Frontal Gyrus, pars triangularis; post-IFG: Inferior Frontal Gyrus, pars opercularis; vmPFC: Ventromedial Prefrontal Cortex; dmPFC: Dorsomedial Prefrontal Cortex; ACC: Anterior Cingulate Cortex; PCC: Posterior Cingulate Cortex; SMG: Supramarginal Gyrus; AnG: Angular Gyrus; pMTG: posterior Middle Temporal Gyrus; STG: Superior Temporal Gyrus; MTG: Middle Temporal Gyrus; ITG: Inferior Temporal Gyrus; FuG: Fusiform Gyrus; TP: Temporal Pole; PHG: Parahippocampal Gyrus; Hpc: Hippocampus.

### 2.2 Controls

Ten controls [7 females; age range 59–82, M = 70.8 years (SD = 7.5); education leaving age = 18.1 (SD = 12.8)] took part in the study. None of the controls had a history of psychiatric or neurological disorder. They were matched to the patients on age [t(17) = −1.77, *p* = .095] and years of education [t(12.7) = −1.71, *p* = .111].

### 2.3 Open access and declarations

The conditions of our ethical approval do not permit public archiving of brain data, because participants did not provide sufficient consent. Researchers who wish to access the data should contact the Research Ethics Committee of the Department of Psychology, University of York, or the corresponding author. Sufficient data to replicate all results reported in the paper will be released to researchers, subject to the approval of the Research Ethics and Committee of the Department of Psychology, University of York, when this is possible under the terms of the GDPR (General Data Protection Regulation EU 2016/679). Behavioural data are provided in Open Science Framework (https://osf.io/sjchk).

Digital study materials (i.e., pictorial stimuli, experimental scripts and stimuli ratings as described in the following sections) are provided on Open Science Framework (https://osf.io/68rxh). The background neuropsychological materials are not provided on OSF since these included published and copyrighted tests, and because they were administrated as ‘paper and pencil tests’. Researchers who wish to access these materials should contact the corresponding author.

Codes of analyses (https://osf.io/w8gq4) as reported in the following sections are provided on Open Science Framework.

No part of the study procedures and analyses was pre-registered prior to the research being conducted. All manipulations and measures of this study are reported in the following sections.

## 3 Background neuropsychology

### 3.1 Non-semantic tests

Data for individual patients are shown in Supplementary Table 2. The “cookie theft” picture description (Goodglass & Kaplan, 1983) revealed non-fluent speech in half of the patients. Word repetition (PALPA 9; Kay, Lesser, & Coltheart, 1992) was also impaired in four patients out of nine. Executive/attentional impairment was seen in seven of the nine patients across four tasks: Elevator Counting with and without distraction from the Test of Everyday Attention (Robertson, Ward, Ridgeway, & Nimmo-Smith, 1994); Ravens Coloured Progressive Matrices (RCPM; Raven, 1962); Brixton Spatial Rule Attainment task (Burgess & Shallice, 1997) and Trail Making Test A & B (Reitan, 1958). This is in line with previous studies which found that deregulated semantic cognition was associated with executive dysfunction in stroke aphasia (Jefferies and Lambon Ralph, 2006, Noonan et al., 2010, Thompson et al., 2018). Digit Span was impaired in all patients, while six out of nine had spatial spans in the normal range. The patients showed normal performance in the Face Recognition task from the Wechsler Memory Scale (WMS-III, Wechsler, 1997) which has minimal control demands, confirming they were not amnesic.

### 3.2 Cambridge semantic battery

This assesses semantic retrieval for a set of 64 items across tasks (Adlam et al., 2010, Bozeat et al., 2000), including picture naming, word-picture matching, and verbal and pictorial semantic associations (Camel and Cactus Test, CCT). Patients showed large variability in picture naming, reflecting additional phonological deficits in some cases [percentage correct M(SD) = 62.8% (39.5)]. In contrast, performance was uniformly at ceiling in word-picture matching [M(SD) = 95.7% (5.7)], indicating intact comprehension in tasks with minimal control demands. On the CCT, when associations between concepts had to be retrieved and control demands were higher, there was greater impairment, with no differences across modalities [words M(SD) = 78.6% (17.2); pictures M(SD) = 77.4% (14.4)]. Individual test scores are provided in Supplementary Table 3. Pairwise correlations across the six combinations of these four tasks revealed no significant associations between tasks [*p* ≥ .110]. Only when tasks had the same control demands across different modalities - i.e., during word and picture association judgements – did this correlation approach significance [r = .64, *p* = .066]. This is in line with the findings of Jefferies and Lambon Ralph (2006), who found consistent performance across modalities within the same task (when control demands remained constant) but not between tasks with different controlled retrieval requirements.

### 3.3 Tests of semantic control

Three tasks manipulated the control demands of verbal and non-verbal semantic judgements (see Supplementary Table 3 for individual data; previously reported by Stampacchia et al., 2018).

#### 3.3.1 Ambiguity task

Semantic judgements (60 items) probed the dominant (money) and subordinate (river) meanings of ambiguous words (e.g., bank). These decisions were presented without cues or preceded by a sentence that primed the relevant interpretation for that trial (cue condition: e.g., for money, I went to see the bank manager) or the irrelevant interpretation (miscue condition: e.g., the bank was slippery). There were four response options on each trial. Further details are available from Noonan et al. (2010). All the patients were below the normal cut-off in all conditions. Every individual patient showed better comprehension for dominant than for subordinate interpretations [no cue condition percentage correct: dominant M (SD): 81.1 (11.1); subordinate M (SD) = 53.0 (13.7)]. In addition, every single patient showed additional impairment in accessing subordinate meaning following miscues rather than cues [percentage correct subordinate trials: miscues M (SD) = 44.1 (15.3); cues M (SD) = 72.6 (14.5)]. In a 2 (dominant *vs* subordinate) by 3 (cue, no cue, miscue) by 2 (patients, controls) ANOVA, there were main effects of dominance [F(1,15) = 80.22, *p* < .001] and cueing [F(2,14) = 18.39, *p* < .001] plus interactions of dominance by cueing [F(2,14) = 7.28, *p* = .007], dominance by group [F(1,15) = 48.35, *p* < .001], cueing by group [F(2,14) = 18.19, *p* < .001] and the three-way interaction [F(2,14) = 5.61, *p* = .016; control data from Noonan et al., 2010].

#### 3.3.2 Synonym judgement task

We presented a synonym judgement task (84 trials) from Samson, Connolly, and Humphreys (2007). Trials included strong or weak distractors; e.g., dot with point [target], presented with dash [strong distractor] or leg [weak distractor]. There were three response options per trial. Accuracy was below the cut-off for all patients and poorer when semantically-related but irrelevant distractors were presented [percentage correct: weak distractors M (SD): 67.7 (11.4); strong distractors M (SD): 45.8 (13.5)]. In a 2 (strong/weak distractors) by 2 (patients, controls) ANOVA, there was a main effect of condition: F(1,15) = 10.19, *p* = .006 and an interaction with group: F(1,15) = 20.81, *p* < .001 (control data from Samson et al., 2007).

#### 3.3.3 Object use task

An object use task (74 items) involved selecting an object to accomplish a goal (e.g., bash a nail into wood), with all items represented as photographs, from Corbett et al. (2011). The target was either the canonical tool, normally used to complete the task (e.g., hammer), or a non-canonical option that could be used instead (e.g., brick), presented among a set of five unsuitable distractors. All patients were poorer at selecting non-canonical targets [percentage correct: canonical M (SD) = 91.9 (7.9); alternative M (SD) = 58.6 (19.5); t(8) = 7.72, *p* < .001] and impaired compared to controls [t(8.4) = 5.87, *p* < .001; control data from Corbett et al. (2011), and not collected for the canonical condition given near-ceiling performance]. One single patient (P5) was not below the normal cut-off in the non-canonical condition; however this case was impaired at the pictorial version of the CCT and consequently still showed evidence of a multimodal deficit.

## 4 Source memory: methods overview

### 4.1 Overview of research questions addressed in each experiment

This section provides an overview of the four experiments to introduce the reader to the main experimental manipulations of this study. More details about the methods, together with the results, are provided below – in separate sections – for each experiment. Experiment 1a examined the role of a spatial cue in ameliorating source memory deficits in SA. During an encoding phase, photos of everyday objects were placed in different coloured boxes. During recollection, participants were asked to decide whether they had seen each item (familiarity judgement). When they recognised items as ‘old’, they were asked which box it had been placed in (source judgement). In the recollection phase, items and sources (i.e., photographs of the coloured boxes) were shown on a computer screen. In Experiment 1a, the boxes were presented in different positions on the screen. In Experiment 1b, the boxes were in the same spatial location as at encoding. In Experiment 2, we retained the spatial cues and examined source memory trials that were congruent or incongruent with knowledge. The stimuli were items that would be purchased in specific shops (e.g., fruits and vegetables and bakery products), presented in a semantically-congruent source (a carrot in a box labelled greengrocer) or a semantically-incongruent source (e.g., carrot in the bakery). We next manipulated the meaningfulness/distinctiveness of the sources using self-reference paradigms. In the encoding phase of Experiment 3, the participant and tester each had a basket, and shopping items were ‘won’ by either person and placed into these baskets. We then assessed item and source memory for self- and other-related items (retaining spatial location as a valid cue). Experiment 4 assessed the memory advantage for self-related items using a classic verbal self-reference paradigm. Personality trait-adjectives were either encoded to reference to the self or an acquainted other (i.e., the Queen) or shallow processed (i.e., judgement about font, e.g., “case” condition); source and item memory were then assessed.

### 4.2 Scoring and analysis

Item and source accuracy were scored using a discrimination index Pr (Snodgrass and Corwin, 1988). This index was preferred to standard measures of accuracy (e.g., percentage correct) because it controls for guessing in the item familiarity task; however, unlike other metrics, like d′, it allowed a direct comparison between item and source memory in Experiments 1–3. Pr was scored as: a) the difference of hits minus false alarms, for item memory; b) the difference between correct and incorrect responses divided by the number of hits, for source memory. Pr varied between 1 and -1, with chance being 0 for 2AFC tasks (Experiments 1–3) and −.33 for 3AFC (source memory decisions in Experiment 4). In Experiments 1–3, ANOVAs were used to assess effects of memory type (item *vs* source) and encoding condition (e.g., congruent *vs* incongruent) by group (patients *vs* controls). In Experiment 4, since the number of response options in item memory (two: yes and no) and source memory (three: case *vs* self *vs* other) were no longer comparable, separate ANOVAs were computed for source and item memory, examining encoding condition (i.e., self *vs* other *vs* case) by group.

## 5 The effect of spatial location source memory (Experiments 1a and 1b)

### 5.1 Rationale

Experiments 1a and 1b examined the role of spatial location in episodic recollection. In Experiment 1a, the location of the boxes at encoding was not maintained on the screen during recollection - preventing participants from relying on spatial cues during source recollection - while in Experiment 1b, the boxes were always presented on the left or right-hand side, during both encoding and retrieval. We expected source memory to be more impaired than item familiarity in SA patients, especially in the absence of spatial cues.

### 5.2 Method

#### 5.2.1 Procedure

**Fig. 2:**
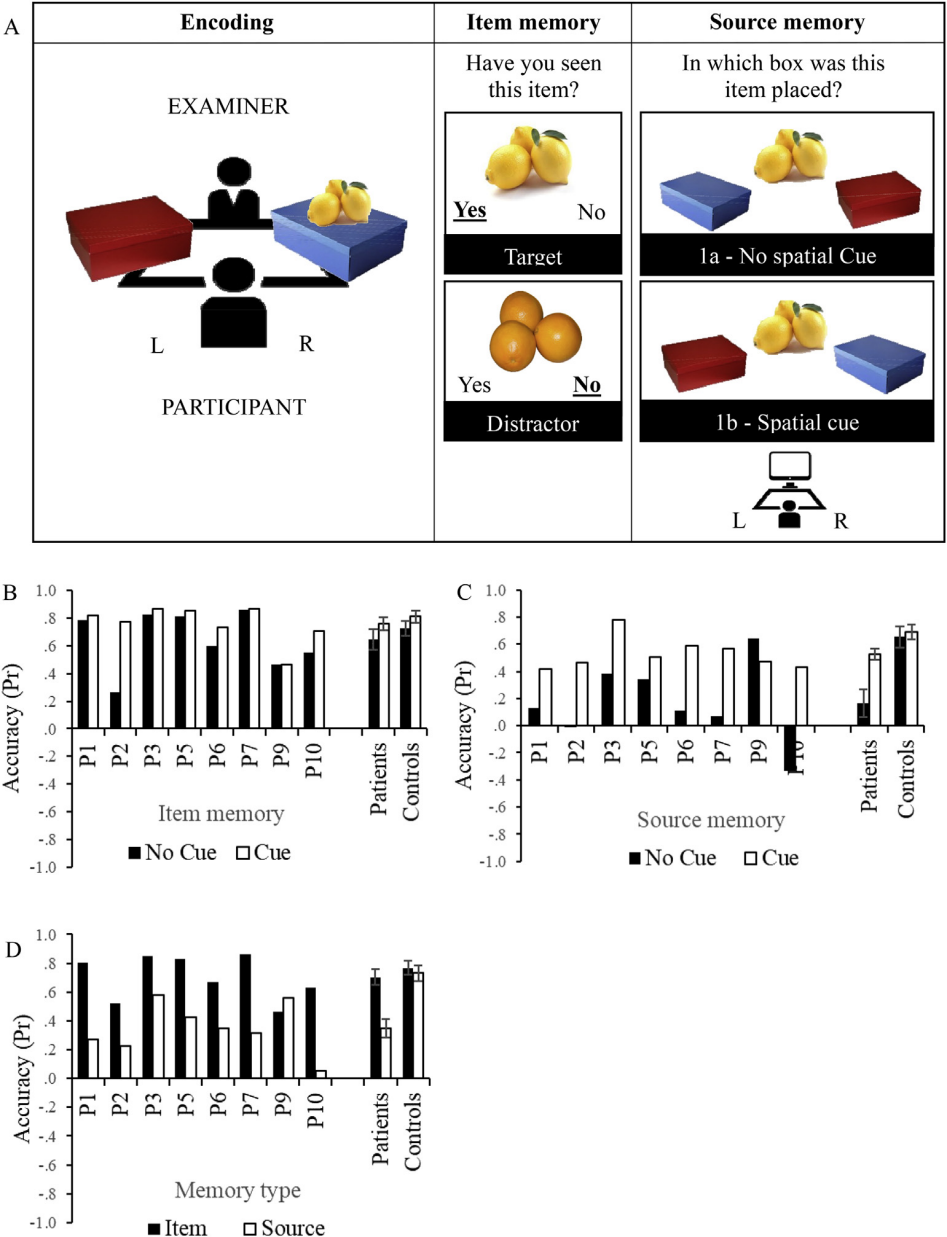
Experiment 1 design and results. A) Schematic of encoding, item and source memory phases of Experiments 1a and 1b. L = participant's left-hand side, R = participant's right-hand side. Both target items (previously presented) and distractors (semantically-related items) were presented during item memory decisions. For items judged as familiar, source memory was tested. During source memory decisions, in Experiment 1a, boxes were randomly allocated to the L or R hand-side, preventing participants from relying on the spatial location at encoding; in Experiment 1b, the position of the boxes at encoding and at retrieval was the same. B) Item memory accuracy during Experiments 1a (No Cue) and 1b (Cue). C) Source memory accuracy during Experiments 1a (No Cue) and 1b (Cue). D) Item and source memory accuracy collapsed across Experiments 1a and 1b. Accuracy is expressed using a discrimination index Pr, with 0 being chance level. Error bars show SE of mean.

A schematic of the task is shown in Fig. 2A. During encoding, a set of 40 shopping items, shown as coloured photographs on 14.5-by-10cm laminated cards, were each presented for around 3 sec, named by the experimenter and placed in a blue or red box in front of the participant. Items were split 50/50 between boxes and the allocation of items to sources was randomized between participants. During a retrieval phase immediately afterwards, coloured pictures of the 40 targets and 20 distractors were presented individually on a laptop screen using E-prime 2.0. Items were randomly assigned to target/distractors between participants. In Experiment 1a (without spatial cues), the position of the boxes on the screen (left *vs* right) was alternated on every trial, such that the location of the target was not systematically related to the location of the source at encoding. In Experiment 1b (with spatial cues), the layout of the boxes on the screen preserved the spatial layout at encoding. The two experiments were administered in separate sessions and Experiment 1a always followed Experiment 1b (the labelling of experiments does not reflect the chronological order of administration and instead the absence and presence of cues). In Experiment 1b, during the study phase, participants were simply instructed to try to remember the items and which box each item was put into. In contrast, in Experiment 1a, they were explicitly told not to rely on the position of the boxes, but on their colour given that later, at retrieval, the position of the boxes on the screen would not match that at encoding. When the item was put into the relevant box, the examiner would narrate “the lemon goes into the blue box”. During the retrieval phase, participants were instructed to indicate for each item, (i) whether the item had been presented previously (selecting “yes” or “no”) and (ii) only for familiar items, which box they had been placed in (selecting the blue or red box). Items remained on screen until the button press, with no time limit for response. This procedure was repeated twice in the no spatial cue condition, and three times in the cue spatial condition, in separate sessions, using different stimuli. This provided 120 trials for analysis in Experiment 1a and 180 for Experiment 1b; this difference is due to participants' reduced availability during testing of Experiment 1a. To ensure that patients comprehended the instructions, Experiment 1b was preceded by practice trials testing item and source memory for 15 items. When the response was wrong, the correct answer was provided along with further explanations until the participant showed evidence of understanding the task requirements. This was not necessary for Experiment 1a given that participants were already familiar with the task.

#### 5.2.2 Stimuli

In Experiment 1a (without spatial cues), the stimulus set comprised of 120 items commonly found in supermarkets, drawn from the following categories: drinks, tinned and canned products, general household and toiletries. In Experiment 1b (spatial cue condition), we used a set of 180 items, including the above categories, plus fruit and vegetable and bakery products. Below, we present an omnibus analysis across all items and conditions. An analysis of data using only the items presented across the two experiments revealed a similar pattern of results (see Supplementary Materials section 1). The list of stimuli is provided in Appendix Tables 1a, b.

### 5.3 Results

We performed a two-way mixed ANOVA, including memory type (item, source), spatial cueing (spatial cue present/absent) and group (patients, controls) as factors. Interactions were explored using separate ANOVAs for patients and controls. Accuracy was lower for the patients [main effect of group: F(1,16) = 7.57, *p* = .014] and for source memory [main effect of memory type: F(1,16) = 28.16, *p* < .001]. There was a memory type by group interaction [F(1,16) = 8.23, *p* = .011] revealing source memory impairment for patients only [main effect of memory type for patients: F(1,7) = 23.45, *p* = .002; and for controls: F(1,9) = 4.29, *p* = .068, Fig. 2D]. There was a main effect of spatial cueing [F (1,16) = 25.87, *p* < .001]; performance was improved if location was a valid cue. This effect interacted with group [spatial cueing by group interaction: F(1.16) = 11.25, *p* = .004], revealing greater benefit from spatial cue for the patients [main effect of spatial cue patients: F(1,7) = 16.87, *p* = .005; controls: F(1,9) = 5.63, *p* = .042]. There were also interactions of spatial cue by memory type [F (1,16) = 6.59, *p* = .021] and memory type by spatial cueing by group [F (1,16) = 8.94, *p* = .009]. The effect of spatial cueing was greater during source than item memory, but only for the patients [memory type by spatial cueing interaction for patients: F(1,7) = 9.22, *p* = .019; and for controls: F(1,9) = .167, *p* = .693]. With the exception of one single case (P9), all patients showed poorer source memory when the spatial cue was unavailable (Fig. 2C). We also explored whether the consistency of box location at retrieval, relative to encoding, had an effect of source accuracy in Experiment 1a. If participants relied on colour features only (not location) – as instructed – spatial consistency between study and retrieval phase should have no effect on source accuracy. A 2 (spatially consistent *vs* spatially inconsistent trials) x 2 (patients *vs* controls) ANOVA looking at source accuracy, revealed a main effect of group [F(1,16) = 15.01, *p* = .001] and no main effect of location consistency [F (1,16) = .22, *p* = .646] nor interaction with group [F(1,16) = .46, *p* = .510].

### 5.4 Summary of Experiment 1

Patients selected to show controlled retrieval deficits in semantic cognition also showed poor source recollection in episodic memory, especially in the absence of strong spatial cues that helped to disambiguate the sources.

## 6 The effect of meaning in source memory (Experiment 2)

### 6.1 Rationale

Experiment 2 examined the role of existing knowledge in source recollection. We presented shopping items within ‘shops’ that were semantically-congruent or incongruent with the category of the item (e.g., fruit and vegetable items were placed either in the greengrocer or the bakery). We anticipated that patients would have greater problems than control participants in retrieving sources that conflicted with background knowledge.

### 6.2 Method

**Fig. 3:**
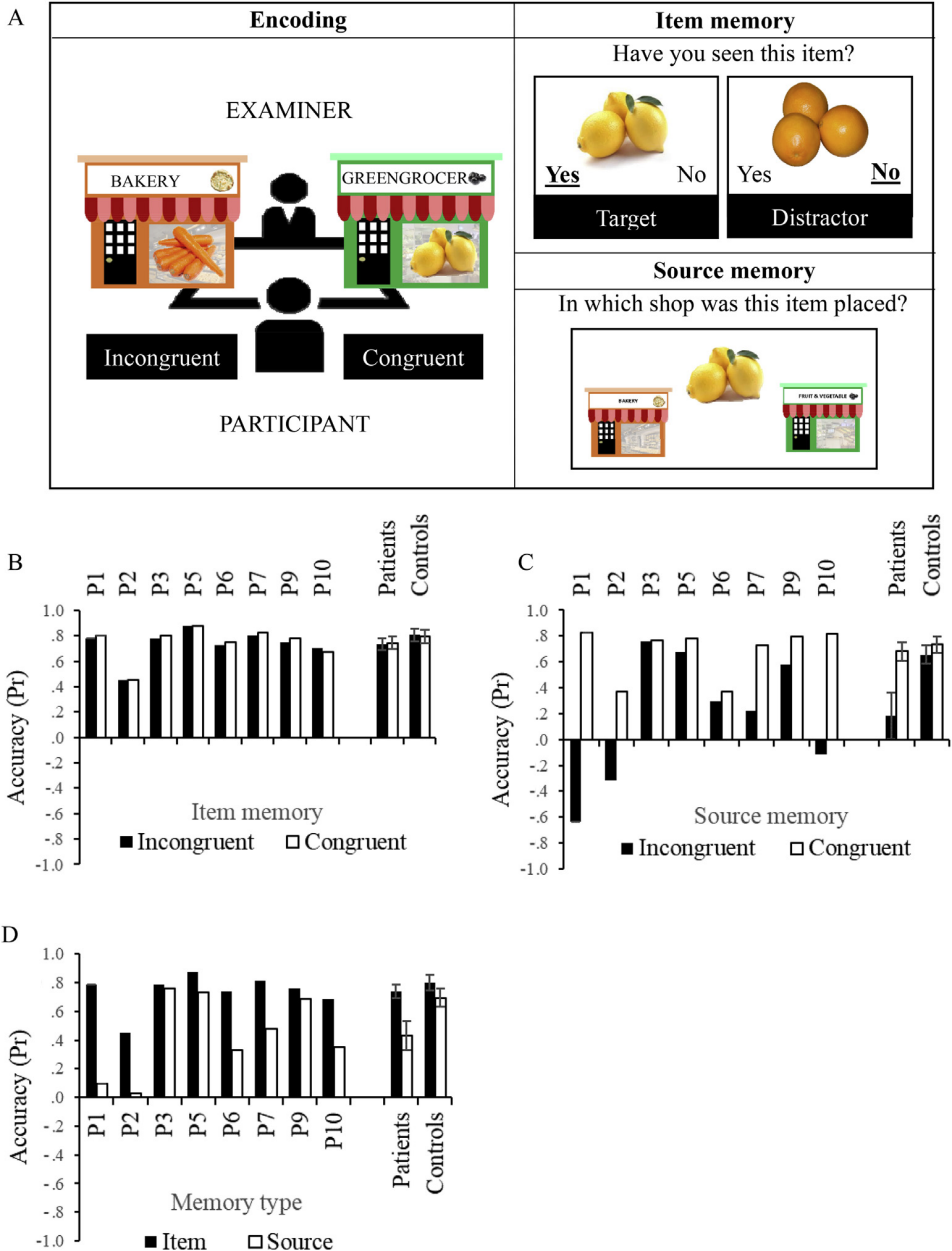
Experiment 2 design and results. A) Schematic of encoding, item and source memory phases of Experiment 2. At encoding, items were either allocated to sources congruent or incongruent with existing semantic knowledge. Both target items (previously presented) and distractors (semantically-related items) were presented during item memory decisions. For items judged as familiar, source memory was tested. B) Item memory accuracy for congruent and incongruent trials. C) Source memory accuracy for congruent and incongruent trials. D) Item and source memory accuracy collapsed across congruent and incongruent trials. Accuracy is expressed using a discrimination index Pr, with 0 being chance level. Error bars show SE of mean.

A schematic of the task is shown in Fig. 3A. Participants were instructed to try to remember a series of shopping items, allocated to one of two shops, represented by boxes labelled with coloured pictures of the store. Participants were warned that items would not be necessarily allocated to the store in which they are usually found (e.g., carrots could be placed into the bakery). During encoding, participants were shown a set of 40 shopping items pictures. Each item was presented for around 3 sec, named by the experimenter and placed in either the congruent or the incongruent shop (20 items per condition). During a retrieval phase, administered immediately afterwards, these target items plus 20 distractors were presented individually on a laptop: participants decided a) whether each item had been presented previously; and b) which shop these familiar items had been placed in. Items remained on screen until the button press, with no time limit for responses. The procedure was repeated twice on separate sessions, so that there were 40 + 40 congruent, incongruent targets and 40 distractors in the analysis. Experiment 2 used the same items as Experiment 1a, and items were randomly assigned to conditions prior to testing each participant. List of stimuli is provided in Appendix Tables 1a, b. All other details of the procedures at encoding and retrieval are as described for Experiment 1. At this stage of testing participants were already familiar with the task requirements (having already done Experiment 1b). To ensure that patients understood the need to indicate the shop in which the item was placed, as opposed to the one in which it is usually found, examples of congruent or incongruent trials were provided. When the response was wrong, the correct answer was provided along with further explanations until the participant showed evidence of understanding the task.

### 6.3 Results

We examined the effects of memory type (item, source), semantic congruency (congruent, incongruent) and group (patients, controls). Interactions were explored using separate ANOVAs for patients and controls. There was no significant difference in overall accuracy across groups [F(1,16) = 3.65, *p* = .074]. Both groups were less accurate during source than item memory [F(1,16) = 25.30, *p* < .001]. There was a memory type by group interaction [F(1,16) = 5.96, *p* = .027], revealing greater impairment for source versus item memory for the patients [main effect of memory type patients: F(1,7) = 16.03, *p* = .005; controls: F(1,9) = 6.75, *p* = .029, Fig. 3D]. There was also a main effect of congruency [F(1,16) = 11.04, *p* = .004], which interacted with group [F(1,16) = 6.56, *p* = .021]: only patients had higher accuracy for congruent versus incongruent trials [main effect of congruency patients: F(1,7) = 8.09, *p* = .025; controls: F(1,9) = 1.16, *p* = .310]. There were also interactions of congruency by memory type [F(1,16) = 10.82, *p* = .005] and congruency by memory type by group [F(1,16) = 5.11, *p* = .038]. The effect of congruency was greater during source than item memory, but only for the patients [congruency by memory type interaction for patients: F(1,7) = 7.06, *p* = .033; and for controls F(1,9) = 2.37, *p* = .158]. This effect of congruency is shown for item memory in Fig. 3B and for source memory in Fig. 3C. All patients but P3 showed poorer source than item memory and higher accuracy during congruent than incongruent source memory trials (Fig. 3C, D). Patients who were semantically more impaired (towards the left-hand side of the graph) systematically chose the wrong source in the incongruent condition (i.e., they assigned items to congruent sources, e.g., carrot in the greengrocer) more often than chance (i.e., accuracy was below 0).

### 6.4 Summary of Experiment 2

Patients with semantic control deficits and PFC lesions were vulnerable to interference from semantic knowledge that was incongruent with recent experience in judgements of episodic memory. This effect was seen strongly in measures of source memory but did not affect recognition of the items themselves. Patients with semantic aphasia are thought to have difficulty controlling competition from strong conceptual representations that are not relevant to the task being performed. Here, they may have failed to control competition between episodic representations of recent events and semantic representations of object meaning when these two sets of representations were in conflict.

## 7 The effect of self-referential processing on source memory (Experiments 3 and 4)

### 7.1 Rationale

Experiments 3 and 4 examined the effect of self-referential processing in source recollection. Self-referential processing is thought to increase the salience and distinctiveness of memories and might therefore decrease the control demands necessary to distinguish between competing sources. However, the effect of self-referential processing on source memory has not been previously explored in patients with semantic control deficits, who have damage to lateral but not medial prefrontal cortex. We expected the patients to show normal self-reference effects (better memory for self-processed items) and, therefore, a higher performance overall, reducing the difference between item and source memory.

In Experiment 3, we instructed participants to remember objects assigned either to themselves or the researcher, using photographs of shopping items as in the experiments above, and tested item familiarity and source memory. This task has been previously shown to promote self-referential processing (Cunningham et al., 2011, Cunningham et al., 2008) and to be associated with medial prefrontal cortex activation (Turk, van Bussel, Waiter, & Macrae, 2011). In Experiment 4, we used a classical self-reference paradigm in which participants were asked to decide whether a personality-trait adjective described themselves or the Queen, or was presented in upper or lower-case letters (focussing attention on surface features of the word). We then performed a surprise memory task including item and source memory decisions.

### 7.2 Experiment 3: method

**Fig. 4:**
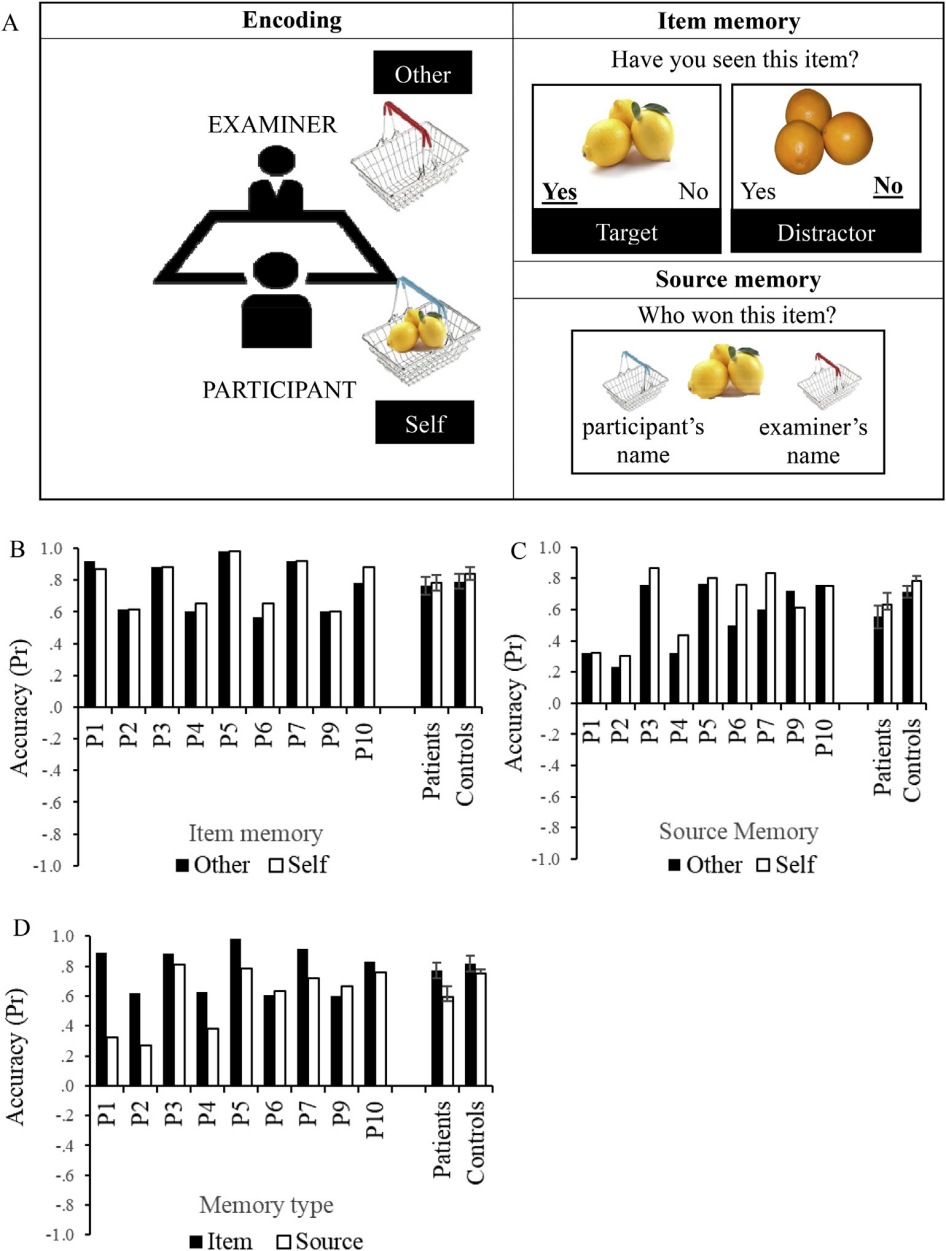
Experiment 3 design and results. A) Schematic of encoding, item and source memory phases of Experiment 3. At encoding items were either placed into the participant's (self) or the examiner's (other) shopping basket. Both target items (previously presented) and distractors (semantically-related items) were presented during item memory decisions. For items judged as familiar, source memory was tested; the participant's and examiner's first names were displayed on screen under the correspondent shopping baskets. B) Item memory accuracy for self and other trials. C) Source memory accuracy for self and other trials. D) Item and source memory accuracy collapsed across self and other trials. Accuracy is expressed using a discrimination index Pr, with 0 being chance level. Error bar show SE of mean.

A schematic of the procedure is shown in Fig. 4A. The task was similar to Experiments 1 and 2, except that the items were placed in two shopping baskets, given to the participant and the researcher. Participants were asked to imagine that they or the researcher had won these items and to try to remember who had received each prize. This experiment used the same items as Experiment 2 (list of stimuli is provided in Appendix Tables 1a, b). All other details of the procedure are as described above. As in previous experiments, practice trials were administered before testing to ensure that patients understood the instructions.

### 7.3 Experiment 3: results

We examined the results using a two-way mixed ANOVA looking at memory type (item/source memory), referent (other, self) and group (patients, controls). Patients and controls did not differ in term of overall accuracy [F(1,17) = 2.35, *p* = .144] and both groups were less accurate during source than item memory [F(1,17) = 12.38, *p* = .003], with no interaction between memory type and group [F(1,17) = 2.95, *p* = .104, Fig. 4D]. There was also a main effect of referent [F(1,17) = 7.32, *p* = .015], which did not interact with group [F(1,17) = .00, *p* = .989] or memory type [F(1,17) = 1.70, *p* = .210]. The three-way interaction of memory type by referent by group was not significant [F(1,17) = .06, *p* = .804]. These results demonstrate a normal self-reference effect in the patients (Fig. 4C).

### 7.4 Experiment 4: method

**Fig. 5:**
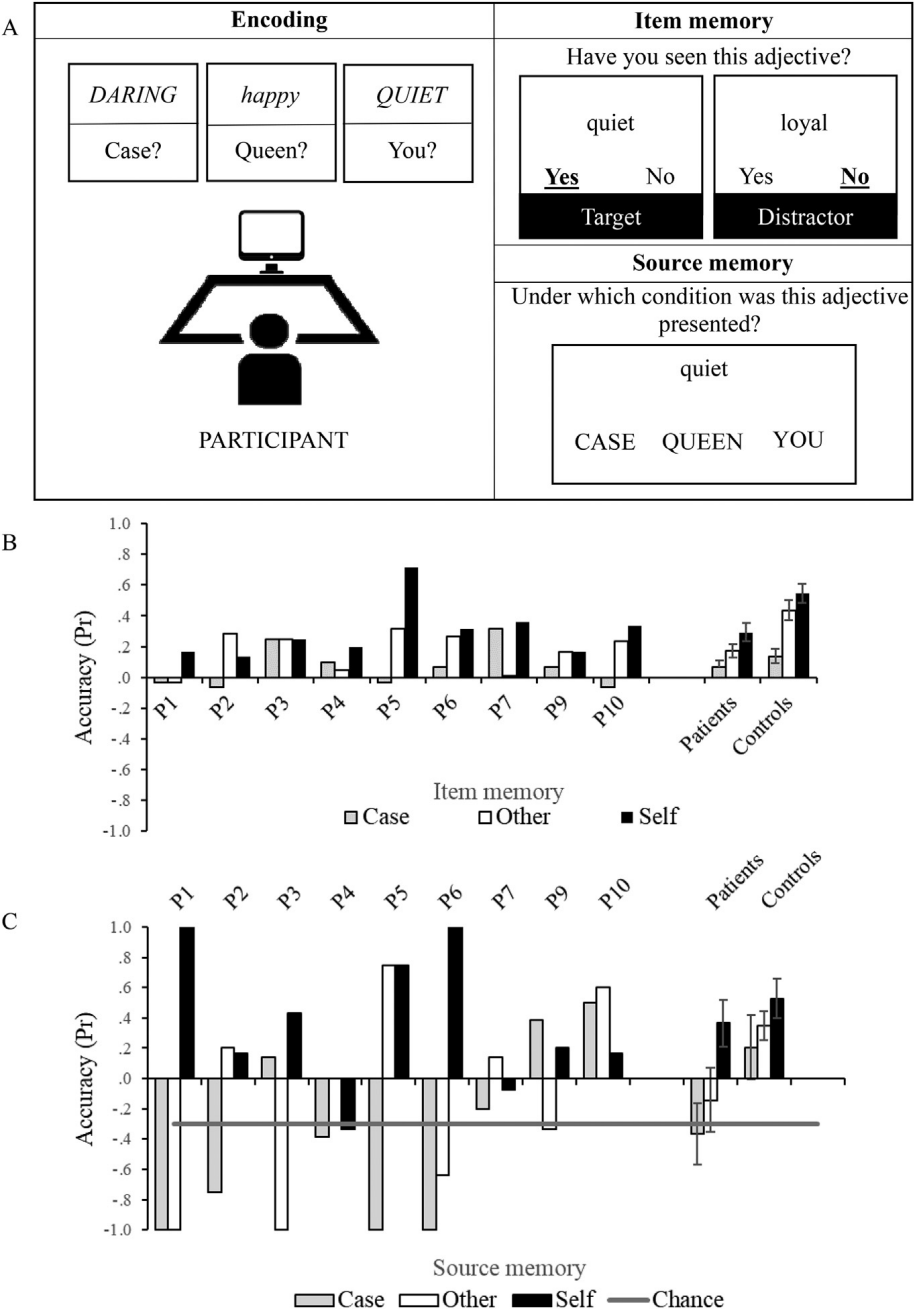
Experiment 4: design and results. A) Schematic of encoding, item and source memory phases of Experiment 4. At encoding participant were asked whether adjective described them (self), the Queen (other) or were displayed in upper or lower case (case). Both target items (previously presented) and distractors (semantically-related items) were presented during item memory decisions. For adjectives judged as familiar, source memory was tested. B) Item memory accuracy for self, other and case trials. C) Source memory accuracy for self, other and case trials. D) Item and source memory accuracy collapsed across self, other and case trials. Accuracy is expressed using a discrimination index Pr, with chance level being 0 for item memory and −.033 for source memory. Error bar show SE of mean.

A schematic of the design procedure is shown in Fig. 5A. During encoding, participants were presented with a list of 60 personality-trait adjectives, read aloud and displayed on the screen, interleaved with 1000 msec periods showing a fixation cross, using E-prime 2.0. Adjectives were allocated to three different encoding conditions, presented in separate blocks of 20 items. During the “self” and “other” conditions, participants decided whether the adjectives described themselves or the Queen, providing a “yes” or “no” response; during the “case” condition, they indicated whether the word was displayed in lower or uppercase letters. Items remained on screen until a response was provided. To make sure that participants understood the referent, an example was provided at the beginning of each block (e.g., Does this adjective describe you? → *kind*) and further explanations were provided when necessary. Participants were not aware at this stage that memory would be tested later. During retrieval immediately afterwards, 60 targets and 60 distractors were presented individually on the screen. Participants decided (i) whether each adjective had been presented previously, by saying “yes” or “no” and (ii) which condition each familiar item had been presented in (by pointing to labels reading “you”, “queen”, “case”). Items remained on screen until a button press, with no time limit for responses. Retrieval was preceded by an example trial, using the adjective presented at encoding (e.g., ‘Was this adjective presented? → *kind*’ and ‘In which condition did it appear? → you, queen, case’). The examiner made sure the participant understood the instructions before starting.

The words were selected from a database of 555 personality-trait adjectives rated for likeability and meaningfulness (Anderson, 1968). They were selected to have neutral valence (likeability from 201 to 401, on a scale from 0 to 600) and high frequency according to SUBLEX (Van Heuven, Mandera, Keuleers, & Brysbaert, 2014). Se lected adjectives were split into two lists matched for likeability, meaningfulness, length and frequency [t < 1, *p* ≥ .352] one used as targets and one as distractors for all participants. At encoding, the assignment of target adjectives to blocks (i.e., self *vs* other *vs* case) was counterbalanced across participants using a Latin square design; the order of block presentation was counterbalanced across participants. At retrieval, items (both targets and distractors) were presented in random order. In order to match visual similarity across conditions, half of the adjectives were presented in upper and lower-case letters during encoding (and in lower case letters during retrieval). A list of stimuli is provided in Appendix Table 2.

### 7.5 Experiment 4: results

ANOVA was used to examine encoding condition (case, other, self) by group (patient, control), for the item and source memory measures separately. Patients showed poorer item memory relative to controls [main effect of group: F(1, 17) = 11.29, *p* = .004]. There was a main effect of encoding condition [F(2,34) = 25.67, *p* < .001], and the interaction with group approached significance [F(2,34) = 2.92, *p* = .067]. Group level contrasts revealed that self-referenced adjectives were better remembered when compared to the case condition in both groups [patients: F(1,8) = 8.88, *p* = .018, controls: F(1,9) = 52.35, *p* < .001]; controls also showed a benefit for other versus case-referenced adjectives [patients: F < 1; controls: F(1,9) = 23.14, *p* < .001, see Fig. 5B]. Source memory was impaired in the patients relative to controls [main effect of group: F(1,17) = 13.57, *p* = .002]. There was a main effect of encoding condition [F(2,34) = 4.09, *p* = .025] and no interaction with group [F < 1]. Contrasts revealed that both self and other-referenced adjectives were better remembered than case [self *vs* case: F(1,17) = 6.42, *p* = .021; other *vs* case: F(1,17) = 4.39, *p* = .051, see Fig. 5C].

### 7.6 Summary of Experiments 3 and 4

In Experiment 3, SA patients showed normal self-reference effects. When sources were self-relevant, they no longer showed source memory deficits, relative to item memory. In Experiment 4, patients again showed the normal benefits of self-referential processing on memory. Self-referential adjectives were better remembered than adjectives relating to someone else, or more shallowly processed words.

## 8 Discussion

We investigated item familiarity and source memory in a sample of semantic aphasia patients who had semantic control deficits and lesions of LIFG, to examine the possibility of parallel impairments of episodic and semantic memory characterised by difficulties overcoming competition from strong but irrelevant representations and a failure to control retrieval in line with the requirements of the task. In particular, we considered whether these individuals would show poor source memory in the context of relatively normal judgements of item familiarity, given that source memory is thought to draw on control processes that resolve competition between similar sources. We also examined whether the source memory impairment reflected the availability of spatial cues at retrieval (Experiment 1), consistency with pre-existing conceptual representations (Experiment 2) and the degree to which the sources were differentiated by means of self-referential processing (Experiments 3 and 4). In this way, the study delineates the circumstances in which retrieval from episodic memory requires control and provides support for a theoretical account in which shared memory control processes shape retrieval from both episodic and semantic memory.

We found that the magnitude of the source memory impairment was related to factors influencing the degree of competition between similar sources. Patients were more impaired at source memory judgements when sources were retrieved in the absence of spatial cues (Experiment 1). Spatial representations may provide a means of differentiating highly similar sources in episodic memory. The patients also showed greater source memory impairment when shopping items were paired with semantically incongruent sources (i.e., carrots in the bakery as opposed to greengrocer; Experiment 2). During congruent trials, source memory reached normal levels in the patients, but in incongruent trials, patients had difficulty disregarding task-irrelevant semantic associations, suggesting a lack of flexibility in the application of existing knowledge to episodic memory. Finally, the memory impairment for photographs of objects was eliminated when the distinctiveness or importance of the source was increased by means of self-referential processing at encoding (Experiment 3). In Experiment 4, using personality trait adjectives, item and source memory were equally impaired in the patients relative to controls, perhaps reflecting the higher language demands of this task. Nevertheless, the patients showed a normal difference between shallow encoding (decisions about upper/lowercase letters) and deep encoding (decisions about self or the queen), suggesting that both meaning-based and self-referential processes were used by patients to separate sources. In patients with SA, representations of space, meaning and self are all thought to be largely intact, while control over retrieval is impaired (see Fig. 1). Consequently, all three of these representational frameworks can differentiate potentially-confusable sources, reducing competition between memories. In addition, this study provides evidence that patients with SA and lesions to LIFG have source memory difficulties, beyond those normally associated with ageing (Chalfonte and Johnson, 1996, Naveh-Benjamin, 2000). Patients had reduced performance compared to age-matched controls and the youngest participant (P10 aged 40) showed one of the biggest differences in performance between item and source memory, especially in the absence of cues that improved performance (see Fig. 2).

This study supports the hypothesis that shared neurocognitive mechanisms support the controlled retrieval of semantic and episodic memories, extending the findings of a previous study, which employed paired-associate tasks in SA patients with LIFG lesions (Stampacchia et al., 2018). The current work shows that similar deficits of episodic memory are observed in aphasia patients with deregulated semantic cognition, even in highly non-verbal tasks. We found several important parallels between the source memory deficits documented here and the semantic impairment previously described for these patients. These are discussed in turn below:

(i) *Multimodal impairment*: Although patients with SA have aphasia consequent on left-hemisphere stroke, they have controlled retrieval deficits that affect both verbal and non-verbal tasks. In the semantic domain, patients with SA show equivalent deficits in accessing associations presented using words and pictures (CCT, Jefferies & Lambon Ralph, 2006) and they have difficulty retrieving non-canonical uses of objects presented as pictures (Corbett et al., 2011), showing that their semantic control deficits are multimodal. Whilst our previous study (Stampacchia et al., 2018) provided evidence of episodic memory deficits on largely *verbal* paired associate tasks in SA, the current study showed that these deficits extended to inherently *non-verbal* source memory tasks, which involved the formation and retrieval of associations between pictures of objects and coloured boxes, shops or people. The multimodal nature of the controlled retrieval deficit in SA, across both semantic and episodic memory tasks, supports the view that shared memory control processes interact with heteromodal semantic and episodic memory representations, which are formed within brain regions such as the ventral ATL and the hippocampus. Both of these brain regions, implicated in semantic and episodic memory respectively, are thought to integrate a wide range of features across modalities, allowing the formation of representations of heteromodal events and concepts (Eichenbaum, 2017, Lambon Ralph et al., 2017).
(ii) *Sensitivity to cues that constrain retrieval*: In semantic memory, patients with SA are highly sensitive to cues that direct retrieval towards relevant features and associations; for example, relevant sentences enable them to access the non-dominant meanings of ambiguous words (Noonan et al., 2010), and pictures of the common recipients of tools (e.g., paper for scissors, or a nail for hammer) facilitate the production of appropriate actions (Corbett et al., 2011). In a similar way, we found that non-verbal contextual cues (i.e., spatial location, Experiment 1b) acted as potent cues in source memory judgements. It appears that in both episodic and semantic memory judgements, SA patients have greater difficulties than healthy controls when the pattern of retrieval required by the task is relatively unconstrained by the information provided, and therefore the need for internally-generated constraint is higher.
(iii) *Difficulty resolving competition*: Previous research has shown that conceptual retrieval in patients with SA is disrupted by semantic distractors that create competition with target concepts (Noonan et al., 2010, Thompson et al., 2018). Similarly, in this study, SA patients' capacity to recall the correct source for a recently-presented item was impaired when semantic knowledge was in conflict with episodic memory (Experiment 2): this semantic congruency effect is likely to reflect competition between the two memory systems. Similarly, Stampacchia et al. (2018) showed that paired-associate learning was vulnerable to semantic distractors that elicited false memories in SA patients. The patients were also more vulnerable than control participants to proactive interference (e.g., competition within episodic memory). Our observation that self-reference could alleviate the patients' episodic control deficits (Experiments 3 and 4) might be explained in a similar way – self-related representations are highly distinctive and potentially more resistant to competition from non-self-related representations.

All of the patients in the current sample had damage affecting LIFG. This brain region shows greater activation during control-demanding semantic tasks, such as when dominant yet irrelevant representations need to be suppressed or when there are many distractors (Badre et al., 2005, Krieger-Redwood and Jefferies, 2014, Krieger-Redwood et al., 2015, Noonan et al., 2013). A parallel neuroimaging literature has linked LIFG, close to the peak overlap in our patient group, to competition resolution in episodic memory tasks (Badre and Wagner, 2005, Dobbins and Wagner, 2005, Kuhl et al., 2007). For example, a classifier trained on the cortical patterns evoked by target and competitor memories in a retrieval induced forgetting paradigm found that pattern suppression for competitors was linked to greater activity in this area (Wimber, Alink, Charest, Kriegeskorte, & Anderson, 2015). The contrast between source and item memory also reveals LIFG activation (Barredo et al., 2015, Dobbins et al., 2002, Dobbins and Wagner, 2005, Han et al., 2012). These findings are highly consistent with a role for LIFG in resolving competition during both episodic and semantic decisions (Badre and Wagner, 2007, Barredo et al., 2015, Burianova et al., 2010, Burianova and Grady, 2007), in line with our results.

The neuropsychological evidence provided in the current study complements this neuroimaging research, since it suggests that LIFG is likely to play a *necessary* role in the control of both semantic and episodic retrieval. In contrast, the activation of LIFG in episodic memory is considered by some researchers to reflect the importance of semantic or linguistic processing in episodic tasks (e.g., Han et al., 2012); as such, LIFG might not make a necessary or critical contribution to controlled episodic retrieval. In contrast with this view, our results showed that a non-verbal source memory task was impaired in patients with LIFG lesions, not only when there was competition between episodic memory and existing knowledge (Experiment 2), but also when non-meaningful sources competed strongly (Experiment 1a). Although our patient sample had relatively large left hemisphere lesions, extending beyond the area of overlap in LIFG, inhibitory transcranial magnetic stimulation (TMS) studies of healthy volunteers provide a test of the causal role of specific brain regions with higher spatial resolution. This research supports the view that LIFG plays an essential role in controlled semantic retrieval (Hoffman et al., 2010, Krieger-Redwood and Jefferies, 2014, Whitney et al., 2011, Whitney et al., 2012). Future TMS research could test the clear prediction emerging from the current work that inhibitory stimulation to LIFG should disrupt controlled retrieval from episodic as well as semantic memory.

Our findings also reveal circumstances in which there is a reduced need for control processes to resolve competition in memory. These effects can be related to the pattern of brain injury in the SA group. The patients' lesions encompass areas involved in semantic control (Fig. 1A, B). In contrast, ventrolateral ATL implicated in heteromodal semantic representation (Fig. 1C) and regions thought to support spatial and self-referential processing (Fig. 1D, E) are preserved. In line with this, the patients showed intact source memory when episodic memory was congruent with existing knowledge, and when spatial and self-related cues were available. The hippocampus and surrounding cortex are thought to support the integration of spatio-temporal features to form unique event memories (see Eichenbaum, 2017 for a recent review). Since these medial-temporal structures are intact in SA patients, the features of events are likely to be bound together relatively normally by hippocampal networks at encoding. At retrieval, distinguishing between similar sources (i.e., the process of pattern separation) may require additional control when events share spatial-temporal features, i.e., they occur within a narrow time window and in similar locations (as in our experiments). Existing semantic representations can facilitate pattern separation when episodic memories are congruent with existing knowledge or schemas (i.e., in Experiment 2, Gilboa & Marlatte, 2017): when sources are non-meaningful (such as in Experiment 1), this process is more prone to error. Additionally, the availability during retrieval of the egocentric spatial configuration present at encoding can act as a potent cue, as it can facilitate the re-instatement of the remaining features of the event memory from its spatial location. Intracranial recordings show that neurons in the hippocampus and entorhinal cortex track spatial configurations (for a review see Moser, Kropff, & Moser, 2008). When these hippocampal-encoded spatial representations are activated by the external environment, the need to control source retrieval using fronto-parietal regions (including LIFG) may be diminished. As such, rTMS to LIFG in healthy individuals disrupts retrieval of abstract words – requiring competition resolution between multiple meanings – only in the absence of contextual cues (Hoffman et al., 2010). Finally, the patients have intact medial cortical structures (Fig. 1A) implicated in self-referential processing (Fig. 1E, De Caso et al., 2017, Macrae et al., 2004, Philippi et al., 2011, pp. 475–481). Self-reference promotes memory in healthy participants (Hamami et al., 2011, Serbun et al., 2011, Symons and Johnson, 1997) and was also beneficial for the SA patients (Experiments 3 and 4). Self-referential processing is likely to reduce competition between memory sources in several ways (see Humphreys & Sui, 2015 for a general discussion). Self-related stimuli have higher salience (see Sui, Liu, Mevorach, & Humphreys, 2015) and higher intrinsic reward when compared with items with no self-relevance (see Sui, He, & Humphreys, 2012). Self-reference is thought to promote the binding of items and sources, even in the face of amnesic and semantic impairment (Sui & Humphreys, 2013). By this view, self-reference acts a form of “integrative glue” that affects coupling between self-representational regions (i.e., ventromedial PFC) and regions implicated in processing of external stimuli and memory (see Sui & Humphreys, 2015 for a review). This would reduce competition between sources with overlapping surface features, ameliorating the effects of control deficits in SA patients.

In conclusion, this study supports the hypothesis that source memory is impaired in SA patients with lesions to LIFG; they had difficulty suppressing irrelevant information when this competed with the correct source, and often failed to resolve competition between sources that lacked distinctiveness. Conversely, self-referential processing, semantic congruency and spatial processing are sustained by intact structures including midline regions such as medial prefrontal cortex (De Caso et al., 2017, Macrae et al., 2004, Philippi et al., 2011, pp. 475–481), ventral ATL (Binney et al., 2010; Visser & Lambon Ralph, 2011; Visser et al., 2012) and hippocampus (Bird and Burgess, 2008, Eichenbaum, 2017). Representations provided by these structures may provide a means of distinguishing between similar sources and therefore compensate for the impaired role of prefrontal cortex in resolving competition during retrieval. This study also has clinical implications, showing that self-reference, spatial processing and semantic congruency may facilitate the accurate retrieval of episodic memories in patients with memory control deficits.

## Funding

The work was made possible by a grant from the Stroke Association (TSA/12/02). The work was also part-funded by the Wellcome Trust [ref: 105624] through the Centre for Future Health at the University of York. EJ was supported by the European Research Council (FLEXSEM – 771863).

## CRediT authorship contribution statement

**Sara Stampacchia:** Conceptualization, Methodology, Software, Formal analysis, Investigation, Writing - original draft, Writing - review & editing, Project administration, Visualization. **Suzanne Pegg:** Conceptualization, Investigation. **Glyn Hallam:** Investigation, Writing - review & editing, Resources. **Jonathan Smallwood:** Conceptualization, Writing - review & editing. **Matthew A. Lambon Ralph:** Conceptualization, Writing - review & editing. **Hannah Thompson:** Conceptualization, Resources, Funding acquisition, Writing - review & editing. **Elizabeth Jefferies:** Conceptualization, Supervision, Writing - review & editing, Project administration, Resources, Funding acquisition.
