# Supplementary material for: Control the source: Source memory for semantic, spatial and self-related items in patients with LIFG lesions
Source: Cortex. 2019 Oct;119:165–83. doi: 10.1016/j.cortex.2019.04.014 (PMC6864601; doi:10.1016/j.cortex.2019.04.014)
Supplement: Multimedia component 1 [file mmc1.docx]

Supplementary materials

Supplementary Table 1. Demographics

| Patient ID | Age | Gender | Education* | Years since CVA | Type of Aphasia |
| --- | --- | --- | --- | --- | --- |
| P1 | 61 | F | 18 | 6 | Global |
| P2 | 78 | M | 15 | 6 | Mixed Transcortical |
| P3 | 60 | F | 16 | 8 | Global |
| P4 | 67 | M | 15 | 23 | Mixed Transcortical |
| P5 | 59 | F | 18 | 6 | Transcortical Sensory/Anomic |
| P6 | 58 | M | 18 | 13 | Transcortical Sensory |
| P7 | 66 | M | 16 | 6 | Broca |
| P9 | 78 | F | 16 | 4 | Anomic |
| P10 | 40 | F | 16 | 7 | Transcortical Sensory/Anomic |
| Mean | 63 | 5/10 females | 16.4 | 8.9 |  |
| SD | 11.5 |  | 1.2 | 5.6 |  |

*Note.* Age at time of testing. *Age left education, CVA: cerebrovascular accident.

Supplementary Table 2. Non-semantic background tests

| Test | Max | Controls Mean (SD) | Patients | P1 | P2 | P3 | P4 | P5 | P6 | P7 | P9 | P10 |
| --- | --- | --- | --- | --- | --- | --- | --- | --- | --- | --- | --- | --- |
|  |  |  | Mean |  |  |  |  |  |  |  |  |  |
| *Non-semantic language tests* | | | | | | | | | | | | |
| PALPA 9 real word repetition (total) | 80 | 73a | 62.1 | NA | **71** | **42** | 75 | 78 | 79 | **1** | 74 | 77 |
| Category Fluency (8) | - | 62a | 38.4 | NA | 26 | **15** | **26** | **14** | **26** | NA | 80 | **57** |
| Letter Fluency (F, A, S) | - | 18a | 7.2 | NA | 2 | **2** | **6** | **3** | **6** | NA | **16** | **9** |
| Cookie theft (words/minute) | - | - | 25.2 | 0 | 18 | 9 | 12 | 60 | 37 | 0 | 54 | 37 |
| *Executive and spatial processing* | | | | | | | | | | | | |
| TEA: counting without distraction | 7 | 4.2a | 4.8 | **4** | 5 | 6 | 5 | **4** | NT | 7 | 5 | 7 |
| TEA: counting with distraction | 10 | 2.6a | 1.8 | **2** | 3 | **1** | **1** | **2** | NT | 7 | **2** | 6 |
| Raven's coloured matrices (total) | 36 | 32.9 (2.41) | 28.0 | 31 | 29 | 31 | **24** | **19** | 30 | 34 | **21** | 33 |
| Brixton spatial anticipation (correct) | 54 | 28a | 23.4 | **21** | **7** | **18** | **26** | **24** | **23** | 31 | 31 | 30 |
| Trial Making Test A (correct) | 24 | 24a | 23.0 | **19** | **22** | **23** | 24 | 24 | **23** | 24 | 24 | 24 |
| Trial Making Test B (correct) | 23 | 17a | 13.7 | **2** | 23 | **16** | **12** | **1** | **5** | 23 | 19 | 22 |
| *Visuospatial processing* | | | | | | | | | | | | |
| VOSP dot counting | 10 | 8a | 9.3 | **7** | 10 | 10 | 9 | 10 | 10 | 8 | 10 | 10 |
| VOSP position discrimination | 20 | 18a | 17.6 | 19 | 20 | **4** | 19 | **17** | 20 | 19 | 20 | 20 |
| VOSP number location | 10 | 7a | 8.9 | 8 | 10 | **5** | 10 | 10 | 10 | 10 | **5** | 8 |
| VOSP cube analysis | 10 | 6a | 8.1 | 8 | 9 | **4** | **4** | 7 | 9 | 10 | 10 | 8 |
| *Wechsler Memory Scale* | | | | | | | | | | | | |
| Digit Span Forward | 9 | 6.82 (0.64) | 4.0 | **0*** | **5** | **4** | **2** | 6 | **4** | **2*** | **3** | **4** |
| Digit Span Backward | 8 | 5.6 (0.97) | 1.5 | **0*** | **2** | **2** | **0** | **2** | **0** | NA | NT | **3** |
| Spatial Span Forward | 19 | 10(3)b | 6.8 | 10 | 5 | 10 | 6 | 5 | **3** | 6 | 7 | 9 |
| Spatial Span Backward | 19 | 10(3)b | 6.8 | 8 | **2** | 10 | 6 | **3** | **3** | 9 | 10 | 10 |
| Face Recognition Immediate | 19 | 10(3)b | 11.1 | 9 | 10 | 12 | 14 | 17 | 13 | 10 | 9 | 6 |
| Face Recognition Delayed | 19 | 10(3)b | 11.9 | 8 | 13 | 11 | 11 | 18 | 13 | 15 | 8 | 10 |

*Note.* Scores are number of correct. a= Normal cut-off; b=WMS Age adjusted scaled score (SD); Bold underlined numbers denotes impaired scores (less than two standard deviations below mean); NT = unavailable for testing; NA = testing was not attempted because patients were non-fluent; TEA = Test of Everyday Attention; VOSP = Visual Object and Space Processing battery. Digit Span: participants were required to immediately retrieve numbers sequences of increased length, in forwards or backwards order. * = For non-fluent patients we used a paper with numbers wrote down during recall. Patients were instructed to point to each number in the same sequence in which was vocally presented by the experimenter. The sheet was not available during the presentation of numbers to avoid the use of spatial strategy for retaining the sequence in working memory. Face Recognition: participants were asked to remember 24 unfamiliar faces, presented one at time. Memory was tested immediately and following a delay of 25-35 minutes (delayed condition); participants identified 24 target faces amongst 48 stimuli, responding either “yes” or “no” to each face.

Supplementary Table 3. Semantic background tests

| Test | Max | Control Mean (SD) | Patient Mean | P1 | P2 | P3 | P4 | P5 | P6 | P7 | P9 | P10 |
| --- | --- | --- | --- | --- | --- | --- | --- | --- | --- | --- | --- | --- |
| *Cambridge Semantic Battery* | |  |  |  |  |  |  |  |  |  |  |  |
| Picture Naming | 64 | 59a | 40.2 | **1** | 61 | **19** | **50** | 60 | **50** | **3** | **56** | 62 |
| Word-Picture Matching | 64 | 62.7a | 61.2 | 63 | 62 | **60** | 64 | **62** | **62** | **52** | 64 | **62** |
| Word CCT | 64 | 60.7 (2.06) | 50.3 | **39** | **43** | **29** | **53** | 59 | **52** | 57 | 61 | 60 |
| Picture CCT | 64 | 58.9 (3.1) | 49.6 | **31** | **44** | **45** | 56 | **45** | 57 | 54 | 53 | 61 |
| *Ambiguity task* | |  |  |  |  |  |  |  |  |  |  |  |
| Miscued dominant | 30 | 30 (0) | 18.0 | **12** | **13** | **13** | **14** | **20** | **19** | **21** | **24** | **26** |
| Miscued subordinate | 30 | 29 (1.20) | 13.2 | **7** | **10** | **14** | **8** | **10** | **15** | **18** | **18** | **19** |
| No cue dominant | 30 | 29.5 (0.54) | 24.3 | **22** | **18** | **24** | **22** | **24** | **26** | **27** | **28** | **28** |
| No cue subordinate | 30 | 28.9(0.64) | 15.9 | **11** | **9** | **14** | **14** | **19** | **17** | **19** | **21** | **19** |
| Cued dominant | 30 | 30 (0) | 23.4 | **23** | **21** | **19** | **22** | **24** | **23** | **23** | **27** | **29** |
| Cued subordinate | 30 | 29.75 (0.46) | 21.8 | **25** | **14** | **20** | **18** | **19** | **28** | **24** | **23** | **25** |
| *Synonym with distractors* | |  |  |  |  |  |  |  |  |  |  |  |
| Strong | 42 | 39.87 (2.23) | 19.2 | **15** | **12** | **13** | **20** | **21** | **23** | **30** | **22** | **17** |
| Weak | 42 | 41.50 (0.53) | 28.4 | **25** | **23** | **29** | **24** | **27** | **30** | **31** | **28** | **39** |
| *Object use* |  |  |  |  |  |  |  |  |  |  |  |  |
| Alternative | 37 | 33.67a | 21.7 | **14** | **13** | **14** | **21** | 34 | **22** | **22** | **26** | **29** |
| Canonical | 37 | 35.9 a | 34.0 | **32** | **31** | **29** | **35** | 37 | **35** | **33** | 37 | 37 |

*Note.* Scores are number of correct; a = normal cut-off, NT = unavailable for testing, Bold underlined numbers denotes impaired scores (less than two standard deviation below mean)

1. Supplementary analysis of Experiments 1a and 1b

An additional two-way mixed ANOVA was performed using only trials overlapping in both Experiment 1a and 1b. Patients showed preserved item memory and impaired source memory [memory type by group interaction: F(1,16) = 30.37, p < .001] and less impaired performance on trials where spatial cues were available [spatial cue by group: F(1,16) = 13.97, p = .002] especially for source memory [although the three way interaction did not reach significance: F(1,16) = 1.031, p = .325]. There was also a main effect spatial cueing [F(1,16) = 11.08, p = .004] and memory type [F(1,16) = 52.01, p < .001].
