# Supplementary material for: Control the source: Source memory for semantic, spatial and self-related items in patients with LIFG lesions
Source: Cortex. 2019 Oct;119:165–83. doi: 10.1016/j.cortex.2019.04.014 (PMC6864601; doi:10.1016/j.cortex.2019.04.014)
Supplement: Multimedia component 2 [file mmc2.docx]

Appendix

Table 1a: List of stimuli Experiments 1a, 1b, 2 and 3

| List 1 | | List 2 | | List 3 | | List 4 | |
| --- | --- | --- | --- | --- | --- | --- | --- |
| Cat. | Item | Cat. | Item | Cat. | Item | Cat. | Item |
| Fruits & Vegetables | apples | General households | air freshener | Tinned and canned items | anchovy fillets | Toys | action figures |
|  | asparagus |  | aluminium foil |  | artichokes |  | ball |
|  | aubergines |  | baking paper |  | asparagus |  | barbie |
|  | baby corn |  | bin bags |  | beans |  | bike |
|  | bananas |  | bleach |  | beef spread |  | board game |
|  | broccoli |  | broom |  | chestnuts |  | boat |
|  | brussels sprouts |  | candles |  | chopped tomato |  | building blocks |
|  | butternut squash |  | canister |  | cockles |  | car |
|  | carrots |  | cleaning cloths |  | corned beef |  | cards |
|  | cauliflower |  | cleaning wipes |  | custard |  | colouring book |
|  | celery |  | cling film |  | evaporated milk |  | crayons |
|  | chillies |  | dishwasher salt |  | green peas |  | dolls house |
|  | courgettes |  | dishwasher tablets |  | hotdog |  | fire truck |
|  | garlic |  | floor cleaner |  | jelly |  | games console |
|  | grapes |  | food containers |  | lentil soup |  | jigsaw |
|  | green beans |  | food bags |  | mackerel |  | kite |
|  | leeks |  | kitchen roll |  | mashed potatoes |  | lego |
|  | lemons |  | laundry liquid |  | olives |  | nurf gun |
|  | melon |  | laundry tablets |  | passata |  | plane |
|  | mushroom |  | light bulb |  | peaches |  | play dough |
|  | onions |  | matches |  | pesto |  | sand pit |
|  | oranges |  | mop |  | pineapple |  | scooter |
|  | parsnips |  | mop pail |  | red cabbage |  | seesaw |
|  | peppers |  | scourer |  | rice pudding |  | skateboard |
|  | pineapple |  | sponges |  | salmon |  | slide |
|  | potatoes |  | surface cleaner |  | spaghetti |  | teddy |
|  | pumpkin |  | tissue |  | spam |  | till |
|  | spinach |  | toilet cleaner |  | sweetcorn |  | train |
|  | strawberries |  | toilet rolls |  | tomato soup |  | video game |
|  | swede |  | washing-up liquid |  | tuna |  | walkie-talkie |
| Bakery products | bagel | Toiletries and Health | aftershave lotion | Drinks | 7up | Clothes | blouse |
|  | baguette |  | antiseptic |  | apple juice |  | bow tie |
|  | biscuits |  | body cream |  | baileys |  | cardigan |
|  | bread loaf |  | body wash |  | beer |  | dress |
|  | bread roll |  | brush |  | blackcurrant juice |  | gloves |
|  | brownie |  | comb |  | champagne |  | hat |
|  | cheesecake |  | conditioner |  | cider |  | hoodie |
|  | ciabatta bread |  | dental floss |  | cognac |  | jeans |
|  | croissant |  | deodorant |  | cola |  | joggers |
|  | crumpet |  | electrical toothbrush |  | dr pepper |  | jumper |
|  | cupcake |  | facial wipes |  | earl grey tea |  | leather jacket |
|  | flapjacks |  | hair colourant |  | flavoured water |  | leggings |
|  | focaccia bread |  | hair spray |  | gin |  | mini skirt |
|  | fruit loaf |  | hand wash |  | ground coffee |  | pencil skirt |
|  | garlic bread |  | interdental brush |  | hot chocolate |  | play suit |
|  | hot cross buns |  | lemsip |  | instant coffee |  | robe |
|  | hot-dog rolls |  | make-up remover |  | lemonade |  | scarf |
|  | italian bread |  | mouthwash |  | orange juice |  | shirt |
|  | madeleine |  | nail polish remover |  | ovaltine |  | shorts |
|  | muffin |  | paracetamol |  | pimms |  | socks |
|  | pain au chocolate |  | plasters |  | red wine |  | suit jacket |
|  | pancakes |  | razors |  | red bull |  | suit trousers |
|  | pitta |  | sanitary napkin |  | rum |  | sweatshirt |
|  | pizza |  | shampoo |  | sparkling water |  | swim shorts |
|  | scones |  | shaving foam |  | tequila |  | swimsuit |
|  | shortbread |  | shower puff |  | tomato juice |  | trench coat |
|  | sliced bread |  | styling gel |  | tonic water |  | t-shirt |
|  | sponge cake |  | toothbrush |  | vodka |  | underpants |
|  | tart |  | toothpaste |  | whisky |  | vest |
|  | tortillas |  | vitamins |  | yorkshire tea |  | waistcoat |

Legend. Cat. = category

.

Table 1b. List of stimuli and sources for each experiment

| Experiment | List 1 | List 2 | List 3 | List 4 | Sources | |
| --- | --- | --- | --- | --- | --- | --- |
| 1a | 🗸 |  |  | 🗸 | Red box | Blue box |
| 1b | 🗸 | 🗸 | 🗸 |  |  |  |
| 2 | 🗸 |  |  | 🗸 | Fruits and vegetables shop^a^ | Bakery shop^a^ |
|  |  |  |  |  | Toy shop^b^ | Clothes shop^b^ |
| 3 | 🗸 | 🗸 | 🗸 |  | Self | Other |

Legend. a = for list 1; b = for list 4

Table 2. List of stimuli experiment 4

| Target Adjectives | List 1 | List 2 | List 3 | Distractors Adjectives |
| --- | --- | --- | --- | --- |
| indifferent | other | self | case | unhappy |
| silly | other | self | case | compulsive |
| indecisive | other | self | case | worrying |
| crafty | other | self | case | withdrawn |
| critical | other | self | case | impractical |
| self-conscious | other | self | case | fearful |
| inexperienced | other | self | case | preoccupied |
| theatrical | other | self | case | unpopular |
| naive | other | self | case | passive |
| shy | other | self | case | unskilled |
| impulsive | other | self | case | tough |
| quiet | other | self | case | silent |
| suave | other | self | case | frivolous |
| painstaking | other | self | case | meek |
| moderate | other | self | case | dissatisfied |
| convincing | other | self | case | resigned |
| definite | other | self | case | undecided |
| comical | other | self | case | lonely |
| direct | other | self | case | stern |
| artistic | other | self | case | rebellious |
| sad | self | case | other | conventional |
| tense | self | case | other | daredevil |
| timid | self | case | other | cunning |
| withdrawing | self | case | other | extravagant |
| old-fashioned | self | case | other | strict |
| dependent | self | case | other | lonesome |
| eccentric | self | case | other | authoritative |
| ordinary | self | case | other | emotional |
| restless | self | case | other | average |
| unpredictable | self | case | other | blunt |
| changeable | self | case | other | hesitant |
| excitable | self | case | other | aggressive |
| bold | self | case | other | outspoken |
| meticulous | self | case | other | forward |
| proud | self | case | other | methodical |
| daring | self | case | other | shrewd |
| dignified | self | case | other | cautious |
| candid | self | case | other | unconventional |
| careful | self | case | other | persistent |
| orderly | self | case | other | prudent |
| demanding | case | other | self | reserved |
| sarcastic | case | other | self | excited |
| gullible | case | other | self | sensitive |
| troubled | case | other | self | normal |
| radical | case | other | self | subtle |
| anxious | case | other | self | fearless |
| opinionated | case | other | self | systematic |
| forceful | case | other | self | mathematical |
| unlucky | case | other | self | righteous |
| solemn | case | other | self | sentimental |
| conservative | case | other | self | sophisticated |
| innocent | case | other | self | obedient |
| deliberate | case | other | self | quick |
| satirical | case | other | self | persuasive |
| lucky | case | other | self | disciplined |
| objective | case | other | self | idealistic |
| serious | case | other | self | religious |
| confident | case | other | self | social |
| philosophical | case | other | self | precise |
| fashionable | case | other | self | scientific |

Notes: List 1, 2 and 3 denotes the different allocation of adjective to encoding condition (e.g. self vs. other vs. case). Participants were randomly assigned to one of the three list.
